# 

**Published:** 1879-04-20

**Authors:** 


THE Association of Medical Editors will meet in Atlanta, on Monday evening, May 5th, preceding the assembling of the American Medical Association. We anticipate much pleasure in meeting with, and forming the acquaintance of our brethren of the quill.

